# Collectanea Medica

**Published:** 1812-05

**Authors:** 


### COLLECTANEA MEDICA,

CONSISTING OF

### ANECDOTES, FACTS, EXTRACTS, ILLUSTRATIONS, QUERIES, SUGGESTIONS, &c.

RELATING TO THE

*History or the Art of Medicine, and the Auxiliary Sciences.*

"MRS. KENNON, midwife to the Princess Dowager of Wales, a woman of great reputation in her profession, offered to the College of Physicians, through the medium of Dr. Frank Nichols, physician to George II. 1000l. to endow a professorship of midwifery in London, in order to teach women the practice, and to obviate the necessity of employing men."—*Dr. Trusler's Memoirs of his own Life.*

Dr.

Dr. Frank Nichols was a very likely man to advocate and support Mrs. Kennon's proposal: he had taken a pique against the accoucheurs, and was the author of a satirical pamphlet against them, called "The Petition of the unborn Babes to the Censors of the Royal College of Physicians." This is now a very scarce pamphlet.

The *Gentleman's Magazine* for March 1812, p. 205, in a list of collectors of natural curiosities, mentions, that "Mrs. Kennon (the late Queen Caroline's midwife) died in 1755; her collections were sold at Langford's in 1756." Of what did her collections consist?

---

"The muscular coat of the bladder is its outermost, and seems to be just a thin layer of radiated muscular fibres from a centre, the neck of the bladder. I say, radiated, because, in a paper which Dr. Douglas laid before the Royal Society, he endeavors to prove, that the muscular coat is composed of two muscles, one longitudinal and one oblique, which he demonstrated accordingly to the Royal Society upon a human bladder.

"It was a notion he had long entertained, and, as I then dissected with him, I found there was no pleasing him, except the bladder were shewn in this form; and, if the two muscles were not very distinct, we used generally to make them so, by scraping together a few of the fibres with the point of the knife, and thereby making the space between the two muscles the more visible: at the same time that these fibres, we had thus scraped together, put on the appearance of the edge of the muscle. This very bladder that the doctor laid before the Royal Society, I dissected, and confess I made the appearance of two distinct muscles with my knife, in the manner I have mentioned."—*Dr. Hunter's Lectures, MS. taken in short-hand, Feb.* 20, 1752.

---

There is a custom very prevalent among some women who have recovered from the state of childbed, of *going up stairs* before they venture to *come down;* so that many who lie-in on the second floor, go into the garret before they come into the drawing-room; and those who inhabit garrets, step first upon a stool, and from thence upon a chair, before they will trust themselves down stairs. I have asked many the reason of this superstitious custom, but have never found any one who could explain it; the answer always is, that they have been told it is unlucky to come down before they have gone up stairs.

The original intention of this custom was probably to try the patient's strength. If she was well enough to go up stairs, there could be little doubt of her ability to come down; down; but it is possible that she might without much difficulty come down the stairs, without having strength enough to ascend them again.

---

"Guttural tumors are known in Hindoostan by the name of *Ghaigha*, and in Nepaul by that of *Ganoo*. The *Ganoo* appears to be the same with the *Goitre* of the Alps. These Goitres in Nepaul are believed by many of the inhabitants to be an effect of imagination in their pregnant women, who are constantly exposed to the disgusting sight of the protruberant pouches of monkies, with which the sacred grove of *Gorja-sirre* swarms, and which it would be thought an act of the greatest impiety to dislodge."—*Kirkpatrick's Account of the Kingdom of Nepaul.*

---

*Fourcroy and Wedgewood.*

In the excellent "Elements of Natural History and Chemistry," by M. Fourcroy, there is a most extraordinary instance of want of information on the part of this ingenious chemist. "An instrument (he says, vol. i. p. 152, 1788,) capable of indicating with exactness the high degrees of heat, would be an acquisition of great value and importance. We are assured that such an instrument has been constructed in England. *It consists of a very acute-angled cone*, on which a ring of the same matter is occasionally placed. The contraction* of the dimensions of the cone by heat causes the ring to sink to a position nearer the base according to its intensity. This ingenious instrument is yet unknown in France." This is a strange misrepresention of Mr. Wedgewood's thermometer, and but for the above passage it would hardly be believed that M. Fourcroy could have been so ill-informed respecting an invention (of which we so well understood the importance and felt the want) that was described at large in the Philosophical Transactions so long ago as 1782.

---

*Mermaid of Haerlem.*

In the 23d volume of the Memoirs of the Phil. Soc. of Haerlem, Mr. Vosmaer, director of the collection of Nat. Hist. there (1786) belonging to the Prince of Orange, examines, in a memoir expressly on the subject, this extraordinary history. Several of the oldest historians of Holland relate, as an undoubted fact, that in 1403, after a violent storm, in which the *Zuider Zee* had inundated the adjacent

---

* The *contraction* of the cone by *heat* we suppose to be a mistake. As it appears a quotation from Fourcroy, we insert it as we receive it, and leave the correction to our correspondent.—EDITORS.

country, country, some milkwomen from Edam, while crossing the lake near that town, called *Purmeer*, discovered a woman swimming, entirely naked, but discolored with mud and slime. After recovering from their surprise, they rowed up to her, and drew her by force into their boat. She was carried first to Edam, and afterwards to Haerlem, where she was taught to spin; but she always shewed an inclination to return to the sea. In Haerlem she lived several years, and, when she died, was buried in consecrated ground, because she had, of her own accord, shewn marks of reverence to a crucifix. She is said to have had a language of her own, which was, however, unintelligible; nor was she capable of learning the Dutch. This account, extraordinary as it may appear, is confirmed by two very old paintings, one at Haerlem, and the other in the Admiralty Court at Edam; and by a stone statue in the front of one of the gates of the latter city, with an inscription commemorating the event.

By these and several other evidences produced in this memoir, M. Vosmaer considers the fact as sufficiently established, and concludes that this supposed *Mermaid* was an idiot, who probably was deaf and dumb, and had fallen into the sea from some ship that had been wrecked upon the coast. He conjectures also that she might have the singular property of floating long on the water, which practice might render pleasing to her. In support of this opinion, he quotes instances from several writers, of persons endued with this natural levity; and, from one Leegwater, a Dutch author, gives an account of a man who could remain three quarters of an hour under water, and while there not only peeled pears and eat them, *but also played on the hautboy.* Allow Mynheer Leegwater his submarine hautboy player, and no doubt will remain about the mermaid of Haerlem.

---

### *Reception of Vaccination by the Five Nations.*

Dr. Jenner's Treatise on Vaccination was delivered to the council of the Five Nations, by Mr. Claus, with the following address; and, in return, the council presented him with the letter which follows, accompanied by the Wampum Belt, the usual token of friendship with this people, which are now in Dr. Jenner's possession.

Speech of William Claus, Esq. Deputy Superintendent-General of Indian Affairs, to the Five Nations, assembled in council at Fort George in Upper Canada, 8th November, 1807.

Brothers of the Five Nations,—Early in May last, his Excellency Lieutenant-Governor Gore took every possible means to introduce the vaccine inoculation among your tribes, tribes, but, owing to your people being then out on their hunt, it did not take place.

When, on public business here about a month after, I spoke to you again, and strongly recommended to your serious consideration, the introducing among your people this valuable discovery, the want of which you soon afterwards felt very severely in the loss of one of your chiefs, Oughguaghga John.

Brothers,—I have now the great satisfaction to deliver you a book sent to you from England by that great man Dr. Jenner, whom God enabled to discover so great a blessing to mankind; it explains fully all the advantages derived from so great a discovery.

I, therefore, brothers, at his request and in his name, present this book to the Five Nations, as a token of his regard for you and your rising generation, by which many valuable lives may be preserved from that most dreadful pestilence the small-pox.

A true copy. (Signed) Wm. Claus, D. S. G. I. A.

Speech of the Five Nations assembled in council at Fort George in Upper Canada, to Dr. Jenner, London, on the 8th of November, 1807.

Present.

Lieut. Col. Proctor, 41st regt. commanding the garrison,
Wm. Claus, Esq. deputy superintendent-general of Indian affairs,
Lieut. Saunders, 41st regt.
Lieut. Fowler, 41st regt.
Ensign Bullock, ditto,
W. J. Chew, storekeeper, Ind. dept.
David Price, Benjamin Fairchild, } Interpreters.

Brother,—Our father has delivered to us the book you sent to instruct us how to use the discovery which the Great Spirit made to you, whereby the small-pox, that fatal enemy of our tribes, may be driven from the earth. We have deposited your book in the hands of the man of skill, whom our great father employs to attend us when sick or wounded.

We shall not fail to teach our children to speak the name of Jenner, and to thank the Great Spirit for bestowing upon him so much wisdom and so much benevolence.

We send with this a Belt and String of Wampum, in token of our acceptance of your precious gift; and we beseech the Great Spirit to take care of you in this world and in the Land of Spirits.

| Chiefs' Names. | Tribes' Signatures. | Interpretation of the Names. | Nations. |
|---|---|---|---|
| Dewadtharanegea, |  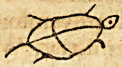 | Two pointed arrows, | Mohawks. |
| Dekayonwagegh, |  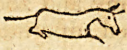 | Two Wampum belts, | |
| Aigowane, |  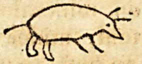 | Clear sky, | Onondagas. |
| Auneai, |  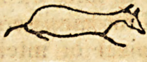 | Feather on his head, | |
| Cosscouete, |  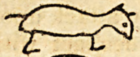 | Moving a tree with brush and planting it, | Senecas. |
| Onindaki, |  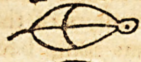 | Town destroyer, | |
| Caugheaw, |  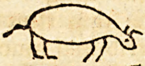 | Raven, | Oneidas. |
| Ussweghtagehte, |  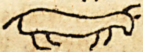 | Belt carrier, | |
| Sawesyewathaw, |  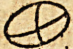 | Disturber of Sleep, | Cayougas. |
| Eajawgehete, |  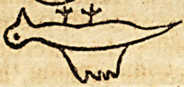 | Fish carrier, | |

### *Some Account of the German Universities.*

The old and once celebrated universities of Germany are tumbling into pieces like the political institutions of the continent of Europe. Within these last two years, accounts have reached us of the universities of Helmstadt, Altdorf, and Rinteln, having expired, and many others are fast approaching towards the same fate. Grieswald and Erfurt are nearly deserted, and Halle seems likely to be eclipsed by the splendid endowments of the newly arranged university at Berlin. The king of Prussia has devoted his attention to the revival of learning, and the arts and sciences, in his capital. He has given a royal palace for the purpose of forming spacious class-rooms for lectures; and he has established galleries for works of art, and museums of natural history. Professors and superintendents have been invited from the neighbouring universities, and medicine, like a wax nose, is to be moulded and fashioned into some new form, to attract the homage of students to the banks of the Spree. Reil has been induced to quit Halle for an appointment and salary adequate to his merit—he presides over the medical department, and was to commence his lectures this present winter. Hufeland, who has been resident at Berlin for several years, is also to deliver lectures on some branch of medicine and physiology. Bernstein is to teach surgery, Hermbstaedt chemistry, Willdenow botany, and Rudolph comparative anatomy and zoology. Professor Weiss, from Leipsic, is appointed superintendent of the mineral cabinet, and to lecture on mineralogy. Gottingen continues to flourish, we are told, under the auspices of king Jerome; and Halle is said to have experienced the patronage of his new majesty and his ministers in a very distinguished manner. This last piece of intelligence interests us as Britons, watching over any attack upon public liberty; and as men of science, who recollect with pleasure their former acquaintance with the members of that once free and celebrated university. Halle has long been distinguished for its eminent professors in the departments of medicine. Stahl, Hoffman, and Gren, contributed much to raise its reputation, which has of late years been well supported by the talents of Reil, Loder, Sprengel, Gilbert, and others. We hope her star will not be eclipsed by being forced into another sphere of motion. Whilst Halle remained under the government of Prussia, she enjoyed all the privileges of independence, by being left alone; the exertions of the professors were not damped by ample salaries, and the pursuits and opinions of the students were not checked by arbitrary regulations.

In 1802, the total number of students at Halle was estimated at 634; in 1805, the medical students amounted to 100, the students of theology to 200; but the most numerous class studied law, which comprehended the sons of noblemen and rich merchants, as well as lawyers, who were sent there for general education. There were no medical societies at which the young men met at stated times, consequently not much emulation or spirit of inquiry among them, though every new system of philosophy had its advocates and partizans. The examinations for conferring degrees are not limited to any particular time of the year; each student undergoes his examination for a degree whenever he demands it; he only submits to one trial, which lasts two or three hours, and is attended by all the medical faculty. Four years is the usual period of study before any application is made for a degree. The candidate defends a Latin thesis publicly, but this is generally written by one of the professors, and is merely *pro forma*. The session for lectures, both in winter and summer, is like that adopted in our Scottish universities. There was no public hospital attached tached to the university seven years ago; the infirmary, which belonged to the town, for the reception of sick poor, only contained twelve beds. The clinical establishment was founded upon a *nosocomium ambulatorium*, a species of dispensary where patients are admitted and visited at their own houses. Cases were entrusted to the care of senior students, who drew up the history of the disease and the daily reports, which were submitted to the clinical professor, and he superintended the treatment, and occasionally visited the patient. In this respect, Berlin is better qualified for a school of medicine than Halle, for it has a large hospital, and good clinical wards.

Loder's museum, and his lecture-room, are under the same roof with his dwelling-house. He lectures on anatomy, physiology, and surgery; and practises as physician, surgeon, and accoucheur. His anatomical preparations are arranged in exquisitely neat order, in several rooms, according to the structure of hard and soft parts of animal bodies. They are very numerous, and many of the injected preparations singularly happy and beautiful. Many rare and important morbid preparations, from which drawings were made for publication, ought ere this time to have been made known to pathologists in this country, if the unfortunate state of public affairs had not stopped our peaceful communication with Hamburgh and Leipsic. There is another extensive collection of anatomical preparations at Halle, which was begun at Berlin by Meckel the first, and has been increased by the labors of his son and grandson. It occupies three large rooms, and is particularly rich in diseased specimens, which probably the present Professor Meckel will describe in the journal of morbid anatomy that he announced two years ago.

Lectures on chemistry and natural philosophy are given by Gilbert, the learned editor of *Annalen de Physik.* His laboratory is well furnished with chemical apparatus; he has models and instruments of all kinds, many of them costly, and all of them purchased and collected by his own industry. It is to be hoped, after so many years' labor, that it will not be deprived of that reward which is justly due to his indefatigable exertions in his favorite study of chemistry, or want that best and most flattering encouragement which a numerous class of pupils can give to his zeal and ingenuity.

The public library is upon a large scale; the building is handsome, situated on an eminence somewhat inconveniently at one end of the town, with trees planted around it, and a row of busts of celebrated men in front. It contains a multitude of books, but complaints were made by the curators

 of of the difficulty in procuring English scientific works; they are so dear, and so rarely exported; and no less difficulty attends the getting books from Italy; but, in spite of these obstacles, the professors are acquainted, through the means of their reviews and periodical works, with every book that is published in the different nations of the world.

The botanical garden is a small one, but it is scientifically laid out by Sprengel, who lectures with great applause on botany. In our frequent visits to the professors, whose politeness and attention could not be exceeded, we frequently heard the praises of our country, not vaguely, but discriminately, marked. The discovery of vaccination was more than once mentioned as the most glorious work of any age, and they could not help envying England the honor of having Jenner for her son and subject.

Perhaps there have been too many universities in Germany, and it may be serviceable to collect and concentrate the talent of the different states; but we fear the scourge of war will destroy many of the good with the bad; and, though the privileged universities may be better supplied, being fewer in number, with the means of affording a scientific education, yet the destruction of so many seminaries where the lectures, as well as the means of living, were cheap, may cut off many *routineers* from the practice of medicine, whose situation in society is very important, and whose condition has been in Germany of late years so much improved.—*Edin. Jour.*

---

*Desultory Observations concerning certain Vegetable Muscicapæ. By Professor* BARTON, *of Philadelphia.*

A few years ago, I accidentally discovered that the flowers of the *Asclepias syriaca* of Linnæus,* like the flowers of the *Apocynum androsæmifolium*, are endowed with the faculty of catching and retaining flies and various other kinds of insects. I have given some account of this discovery in the Transactions of the American Philosophical Society.†

---

* *Asclepias syriaca* β. of Michaux.

† Vol. vi. part i. No. xvi. p. 79-82. Mr. Sonnini has given the credit of this little discovery to an English naturalist of my name. The respectable French naturalist, speaking of the *Asclepias syriaca*, says, "Une propriété curieuse de ces mêmes fleurs, dont la découverte récente est due au Docteur Barton, de *Londres*, c'est qu'elles attrapent les mouches qui s'y posent attirées par le suc mielleux qu'elles contiennent."—"Plus de soixante mouches furent prises .... sous les yeux de l'observateur *Anglais*, &c."—*Journal de Physique*, &c. tom. lxvi. p. 219.

In

In the course of the present year (1811) I have ascertained that the beautiful *Asclepias curassavica* is also a *Muscipula*, or rather a *Muscicapa*. My observations were made on a small and by no means vigorous plant of this species, which I had raised from seed. These observations convince me that this *Asclepias*, in its native climate, or any where else when adult and vigorous, must be a powerful and even *useful* fly-catcher. It often so completely retains insects, even pretty large house-flies, that they are wholly incapable of disengaging themselves, but perish upon the flowers. Others, hardly more fortunate, escape with the loss of their proboscis, or some of their limbs. Few, perhaps, escape *entirely* uninjured. The mechanism by which *Asclepias curassavica* catches flies, is nearly the same as that by which they are caught in *Asclepias syriaca* β.

As the genus *Asclepias* consists of a considerable number of species, and as all the species are so similarly constructed that there is no good reason to doubt that they are all endued with the power of catching insects, it is easy to perceive what an immense havoc these plants must make of *animal* life, especially in many parts of the United States, where some of the species of *Asclepias* are so numerous that they cover hundreds of acres of ground, in close connexion, especially along the banks of our rivers, in the sandy fields, &c.

In the short paper entitled "Memorandum concerning a new Vegetable Muscipula," which is inserted in the Transactions of the American Philosophical Society, and to which I have already referred, I have, I fear, fallen into an error concerning the *Nerium Oleander*, or common Rosebay. My words are these: "It has long been known that this is a poisonous plant; but I do not know that any person than myself has observed that this fine vegetable proves very destructive to the common house-flies. These insects visit the *Oleander*, in order to drink the fluid secreted in the tube of its flowers. The liquor soon intoxicates them, and very few of those which have gained admittance into the blossom ever return from it. So great is the number of flies destroyed in the course of one season by a single *Oleander*, that I have often thought it would be worth our while to pay more attention, than we yet do, to the cultivation of this vegetable; as, independently of its beauty, it is so well calculated to lessen the numbers of a most common and troublesome insect."

Subsequent and more cautious inquiries have convinced me, that, although the nectareous fluid of the *Oleander* may prove deleterious to flies, yet that the greater number of the

 insects insects which are observed dead or dying, in the flowers of this plant, have been entrapped by the irritability of the *genetalia;* by a mechanism, at least, as truly irritable as that by which insects are detained in the flowers of the different species of *Asclepias*, of *Apocynum*, and other similar plants.

In the course of my inquiries and labors concerning the indigenous plants of the United States, I have had the additional satisfaction of remarking, that one of our grasses is also a *Muscicapa*, or at least a catcher of small insects of various kinds; and, as far as I know, it is the only grass hitherto discovered that is entitled to the name of a *Muscicapa.*

The grass to which I allude is the *Leersia lenticularis* of the late Mr. A. Michaux.* This plant is a native of the marshy grounds of the Illinois country, of Virginia, North Carolina, &c. I do not know that it has been found in Pennsylvania. The glume or corolla consists of two valves, a character which belongs to all the species of the genus *Leersia.* In the *Leersia*, which is the subject of my observations, the glume is of an orbicular form, inclining to lenticular, and is much larger than in any of the other American species that are known to me, or than it is in the *Leersia oryzoides* of Europe and America. The edges of the valves are very distinctly ciliated, or furnished with a number of fine teeth or delicate spinules.

It is this ciliate structure that enables the plant to perform the business of a *Muscicapa.* When a small insect, such as a spider, or a minute fly, insinuates itself between the valves (probably in pursuit of a honeyed fluid), the valves close upon it, the spinules enfolding each other, thus retaining the insect, which, I presume, as seldom escapes as the insect that has been caught by the valvular structure at the ends of the leaves of *Dionæa Muscipula.*

In the *Leersia oryzoides* of Swartz and Michaux (*Leersia virginica* of Willdenow), which is called in the United States "cut-grass" and "sickle-grass," the structure of the glume is very similar to that which I have just described. In particular we observe the ciliated structure, though it is less conspicuous than it is in the *Leersia lenticularis.* No doubt, the former as well as the latter of these grasses is entitled to

* Leersia (*lenticularis*) paniculæ ramulis subsolitariis, ramillis secundariis imbricatim spicifloris: glumis lenticulari-orbiculatis conspicue ciliatis, majusculis.—*Flora Boreali-Americana*, &c. tom. i. p. 39.

the the appellation of a *Muscicapa.* But I have not yet observed insects between the valves of the *Leersia virginica.* I may hereafter inquire more particularly into the subject.

In Georgia and in Florida there grows a beautiful shrub to which the inhabitants have given the name of "fly-catcher." This shrub is the *Befaria paniculata* of Michaux.* The inhabitants collect branches of it, when it is in flower, and hang them up in their rooms, and find the flowers of very great use in ridding them of flies: hence its name, which I have mentioned, and the only one, so far as I can learn, by which it is recognised in its native country.

I am not sufficiently acquainted with this plant to say, with certainty, by what power it is that it catches, entangles, or destroys, the flies; whether by a contractile or irritable property, residing in some of the parts and organs of the flowers; whether by the glutinous quality which belongs to the flowers; or whether by a deleterious quality belonging to the nectar of the flower. I may, however, observe in this place, that the *Befaria* is one of the vegetables from which the bees in the countries of Florida, &c. are thought to procure a narcotic or deleterious honey. Still I suspect that the muscipulating faculty of this plant will be found to be owing to a peculiar mechanism; that is, to an irritable power residing in the flowers. But this point remains to be determined by better observations. I have never yet had an opportunity of examining the *Befaria* in a *living* state.

I might here give a long list of vegetables, such as different species of *Rhododendron*, *Kalmia*, *Robinia*, *Silene*, *Lythrum*, which, by virtue of the viscosity upon different parts of their flowers, &c. entangle and destroy small insects. But my business in this imperfect essay is not with *Muscicapæ* of this kind. Yet a more critical inquiry into the use of this viscous matter, in the vegetable economy, by which millions of insects are destroyed in our gardens, green-houses, woods, &c. might deserve the attention of physiologists.

In regard to the *Sarraceniæ*, as I design to make their history, botanical, physiological, and medical, the subject of a distinct memoir, I shall content myself at present by offering a few detached facts and observations concerning these plants.

It is well known that all the species of this singular genus (and I think at least seven species have been discovered in

* Befaria *(paniculata)* ramis hispidissimis: foliis ovali-lanceolatis, glabris: panicula subaphylla, multiflora, glutinosa.—*Flora,* &c. tom. i. p. 280. tab. 26.

North

North America) are inhabitants of the water, or of wet situations. All the species are furnished with tubular or hollow leaves *(ascidia)*, which in the more adult plants are seldom without a considerable number of insects dead or living in them. I do not mean, however, to insinuate that these insects owe their presence in the *Sarraceniæ* to any thing like an *irritable* property residing in any part of the plants; indeed I have not discovered any vestige of peculiar irritability in the constitution of the *Sarraceniæ*. But I think it sufficiently evident that nature has taken some pains (if it be ever allowable to use such language in speaking of the works and operations of Nature) to solicit insects into the ascidia of the species of this genus.

Thus, the flowers of the *Sarraceniæ flava* (the yellow trumpet-leaf or side-saddle-flower of the people of the United States) have a most offensive cadaverous or carrion-like odor. This odor, to speak more properly, seems to reside principally, if not entirely, in the broad peltated stigma of the plant. I think it probable that it is, *in part at least*, this odor which is so potent and diffusible that it is sometimes perceived at a considerable distance from the plant, in a warm and rather confined atmosphere; that it is partly this odor which serves to solicit various kinds of insects about the plant, many of which, before they can reach the stigma, are necessarily entrapped in the ascidia. But I know not what may be the final intention of Nature in giving the peculiar odor which I have mentioned to the stigma of *Sarracenia flava* and some other species of the genus. Those philosophers* who imagine that there are some plants which cannot be impregnated without the aid of insects, may perhaps fancy that they have discovered Nature's ulterior object in the present instance: indeed, I confess myself at a loss to conceive how, in some of the species of *Sarracenia*, the pollen or fecundating influence of the stamens can reach the *upper* surface of the stigma. See in my Elements of Botany, and in other works, the figure of *Sarracenia purpurea*.—And I presume, that it is certainly upon the upper surface of the stigma of these plants that the pollen, or at least its *fovilla*, must be applied, in order to enlarge and render fertile the embryo seeds. In the *Sarraceniæ*, the aid of insects in giving fertility to the germen or ovarium may possibly be necessary. I think it cannot be much less necessary (if at all less necessary) in *Sarracenia purpurea*, than it is said by Mr. Willdenow, Dr. J. E. Smith, and other respectable bota-

* Linnæus, Sprengel, Willdenow, Smith.

nists, nists, to be in *Aristolochia Clematitis.*—See Willdenow's Principles of Botany and of Vegetable Physiology, English translation, pages 316-318. Edinburgh, 1805.* But, although I believe in the doctrine of the sexes of plants as a *general* doctrine, I am compelled by many facts and considerations to doubt whether the impregnation of *any* vegetable is *necessarily* dependent upon insectile aid.† On this curious subject I shall offer something more specific in my "Memoirs on the Origin and Progress of our Knowledge concerning the Sexes of Vegetables."

But there is still a much more considerable and obvious cause of the collections of insects which are so frequently observed in the ascidia of the *Sarraceniæ*. These ascidia, in some if not in all the species of the genus, appear to possess a kind of glandular function, like the true nectaries of a great many plants. A honeyed fluid is secreted or deposited on the inner surface of the hollow leaves near their *faux* or opening; and it is this fluid which allures great numbers of the insects, which they are found to contain, into the ascidia.

I was entirely unacquainted with this curious economy in the ascidia of the *Sarraceniæ* when I published the first edition of my Elements of Botany; and even when I printed the Appendix (in vol. i.) to the second edition of this work. The fact will not be deemed uninteresting by the cultivators of vegetable physiology, since it somewhat enlarges our views of the uses of the ascidia, and may assist us in making a better disposition of these parts among the glands, or

---

* See also Dr. Smith's Introduction to Botany, &c. p. 337, &c. London, 1807. This botanist not only reposes confidence in Willdenow's theory of the insectile impregnation of *Aristolochia Clematitis*, but he thinks it probable that, "for want of some insect adapted to the same purpose in its own country, the American *Aristolochia Sipho*, though it flowers plentifully, never forms fruit" in the British gardens. However it may be in *Aristolochia Clematitis*, I am persuaded that insects are not necessary agents in the impregnation of the germ of *Aristolochia Sipho*, or "Dutchman's-pipe," as it is called in Pennsylvania. I am well acquainted with this plant, and cannot perceive from the structure and disposition of its genitalia any obstacle to the application of the pollen to the female organ. Certainly there is much less difficulty in conceiving how impregnation is effected in this *Aristolochia*, than in hundreds of other plants, concerning the impregnation of which no difficulties have ever occurred in the minds of botanists.

† "The dichogamic plants can be in no other way fecundated than by insects."—*Willdenow*.

glandular-like organs, of vegetables. I have little doubt, moreover, that a more accurate examination of the genera of *Nepenthes*, *Aquarium*, and similar plants, of which the number is not inconsiderable, will render it certain that a honeyed fluid deposited about the opening of the ascidia of these remarkable plants, is in them, as in the *Sarraceniæ*, the *principal* cause of the deposits of insects which the tubular leaves are so generally found to contain. Let me add, that in the American plants, of which I have been speaking, the honeyed fluid may often be very distinctly seen; though sometimes, especially in the warmer weather, it is only to be discovered by the *taste*.

Many insects, as well as some other animals, are found in the ascidia of the *Sarraceniæ*, *Nepenthes*, &c. which do not appear to have been solicited thither either by the cadaverous odour of the flowers, or by the honeyed fluid about the opening of the tubular structure. In the *Sarraceniæ* we find species and sometimes large species of *gryllus*, or grasshoppers, of *gyrinus*, &c. And Rumphius, speaking of *Nepenthes distillatoria*, informs us that "various little worms and insects crawl into the orifice and die in the tube, except a certain small *squilla* or shrimp, with a protuberant back, sometimes met with, which lives there." Smith's Introduction, &c. pages 197, 198. Dr. Smith has "no doubt, that this shrimp feeds on the other insects and worms, and that the same purposes are answered in this instance as in the *Sarraceniæ*."

It is certain, that a remarkable instinct directs some species of insects to visit the ascidia of different species of *Sarracenia*, principally, if not entirely, for the purpose of depositing their eggs or larvæ in them. We often see some of the larger species of *Musca* and *Tabanus* sitting upon the edges of the openings of the ascidia of *Sarracenia flava* and *Sarracenia purpurea*. We soon find that they do not come here, as do the majority of insects, for the purpose of sipping the honeyed fluid from the ascidial glands. The mother-insect, carefully poising herself upon the brim of the ascidium, emits from her uterus into the tube one or more larvæ, which immediately betake themselves to the lower part, where, meeting with abundance of good food, they rapidly increase in size and in strength.

I must give to Dr. Smith the credit of having preceded me in making, or at least in recording, an observation nearly allied to the one which I have just detailed. "An insect of the *Sphex* or *Ichneumon* kind," as far as he could learn from the description communicated to him, "in the Botanic Garden at Liverpool," "was seen by one of the gardeners to drag several large flies to the *Sarracenia adunca*, and, with some difficulty forcing them under the lid or cover of its leaf, to deposit them in the tubular part, which was half filled with water. All the leaves, on being examined, were found crammed with dead or drowning flies." "The *Sarracenia purpurea*," adds Dr. Smith, "is usually observed to be stored with putrefying insects, whose scent is perceptible as we pass the plant in a garden" This I have not observed: and perhaps the odour spoken of may be that exhaled from the flowers, or *genitalia*, of the plant. "Probably (the learned English botanist goes on to observe) the air evolved by these dead flies may be beneficial to vegetation, and, as far as the plant is concerned, its curious construction may be designed to entrap them, while the water* is provided to tempt as well as to retain them. The *Sphex* or *Ichneumon*, an insect of prey, stores them up unquestionably for the food of itself or its progeny, probably depositing its eggs in their carcases, as others of the same tribe lay their eggs in various caterpillars, which they sometimes bury afterwards in the ground. Thus a double purpose is answered; nor is it the least curious circumstance of the whole, that an European insect should find out an American plant in a hot-house, in order to fulfil that purpose."

I feel much obliged to Dr. Smith for leading me, by the preceding observations, to pay a more critical attention than I might otherwise have done to the œconomy of the *Sarraceniæ* and other ascidial plants. Hitherto, however, I have not observed any species of *Sphex*, or of *Ichneumon*, engaged in the singular business which he has mentioned. And, although I do not doubt that the gardener's statement is in the main correct, yet I incline to think that the supposed *Sphex*, or *Ichneumon*, was an insect of some other family. Future observations will, I hope, enable me to determine this point entirely to my satisfaction.

Although I have no doubt that plants may derive much of their nutriment and strength from the elements of decomposed animal bodies, just as I suppose that innumerable species of animals are nourished by inorganic matter; and, although it is possible that the air evolved by the dead insects in the ascidia of the *Sarraceniæ* and other similar plants may contribute somewhat to the vegetation of these plants, yet I cannot suppose that the final intention of Nature in furnish-

* It is evident, from what has already been said, that it is not the water in, but the honey about and upon, the ascidia, which tempts the greater number of insects to visit these tubes, in the different species of *Sarracenia*. Neither is it the water that retains them.

ing plants with ascidial leaves is to lay up a store of nutritious aliment for the plant.

So far as the species of *Sarracenia* are concerned in this view of the subject, I hardly know any plants to which such storehouses as we are speaking of would be less necessary. For they live in the midst or upon the margin of marshes, where animal matters of various kinds are continually putrefying, and where of course the plants are almost constantly surrounded by an atmosphere of azotic and hydrogen gases.

Again, the wonderful *Dionæa Muscipula*, the extremities of whose leaves entrap, and frequently retain for a considerable time, various small species of insects, resides only in marshy grounds, not much unlike those just mentioned. In this plant, it appears to me to be still more difficult to give a plausible guess about the intention of Nature in constructing these vegetable *Muscicapæ*, than in regard to the *Sarraceniæ*.

If, however, we were acquainted with only the last-mentioned vegetables, with *Nepenthes* and with *Dionæa*, as the only plants, I say, endued with the power, however different the means, of catching or entrapping insects, we might, perhaps, with some degree of reason, imagine that this faculty is, somehow or other, subservient to the business of vegetable nutrition. But never can this idea be extended, with even the faintest shadow of a good theory, to those truly irritable *Muscicapæ*, *Apocynum androsæmifolium*, *Asclepias syriaca*, *Asclepias curassavica*, *Nerium Oleander*, and many others, whose flowers entrap insects or the *parts* of insects. For, in these plants, the whole quantity of animal matter applied to and retained by the plants, in their most vigorous state, is a mere atom in comparison of the volume of living vegetable matter to be nourished. Moreover, the animal matter is applied only to the flowers, which no one imagines to be essentially concerned in nourishing the plant; to the *temporary* organs, and not to those which may be called permanent, the leaves or ascidia.

In making these observations, I have not an eye to the theories or speculations of any particular author. I must confess, however, that I once imagined that *Dionæa Muscipula* itself does receive a part of its nourishment from the insects which it entraps. And Mr. Roth, if I do not mistake, has endeavoured to show that the species of *Droseræ*, so common in the bogs of Europe, &c. are, in some measure, indebted for their growth and nourishment to the insects which they entangle and retain. I have not seen the arguments by which the respectable botanist just mentioned supports his hypothesis.

I must

I must not take my leave of the *Sarraceniæ* without observing, that the ascidia of some (perhaps all) of the species of the genus furnish us with still another beautiful example of what the ingenious author of "The Studies of Nature" would call the "harmonies" of the vegetable and animal kingdoms. For, omitting, at present, all further speculations about the intention of Nature in giving to the species of this genus ascidial leaves, and a capacity of entrapping great numbers of insects, it is a fact, that these leaves become repositories of the food of certain species of birds. It is not uncommon, in the native soils of some of the species of *Sarracenia*, to see great numbers of birds, especially some *Muscicapæ*, or Flycatchers, and other *Passeres*, assembling about these plants, and, by means of their bills, slitting the ascidia in order to get at their favorite food.* This well-ascertained fact will not be deemed incurious in a history of the genus *Sarracenia*; nor unimportant in a history of the instincts and intelligence of the great class of birds. We know much less of the structure and œconomy of the different species of the genus *Nepenthes*, and of the only species of the genus *Aquarium* that has yet been discovered, than we do of the structure and œconomy of the species of *Sarracenia*. But it is highly probable, that the ascidia of

* Linnæus tells us, that the hollow leaf of *Sarracenia purpurea* is a reservoir of water for thirsty little birds. "Folium S. purpureæ in Spec. Pl. descriptum, aquam præbet sitientibus aviculis." *Prælectiones in Ordines Naturales Plantarum*, Edit. Giseke, p. 316. Hamburgi 1792. This is doubtless a mistake. The birds supposed to be sipping water from the ascidium of *Sarracenia*, were, I suppose, engaged in the very different business of eating the insects which the reservoir contained. None but a bird with a very long bill would be able to take the water from the tube. Is it likely, moreover, that any bird should be driven to the necessity of satisfying its thirst from the reservoir of a plant which always grows in wet places, and frequently in waters of a foot or more in depth? for *Sarracenia purpurea* is sometimes seen growing in our cypress swamps, its roots, like those of a *Lemna* or an *Utricularia*, being suspended in the water and totally detached from the earth. If it be true, as is asserted, that the *Trochilus Colubris*, or common Humming-bird, is sometimes seen about the ascidia of the *Sarraceniæ*, we seem safe in conjecturing, that this beautiful bird visits these tubes for the double purpose of sipping the honeyed fluid and of eating the insects. I have elsewhere shown that insects constitute a part, and perhaps a considerable part, of the food of this *Trochilus*.—See the Philadelphia Medical and Physical Journal, vol. i. part i. art. xxiv.

both both *Nepenthes* and *Aquarium** will be found by future botanists and naturalists to serve the same purpose, with respect to birds, as do the *Sarracenia variolaris* and other species of the genus in America.

The large ventricose ascidia of *Sarracenia purpurea* are employed as cups, for holding and conveying water, by the reapers and mowers in some parts of the United States, particularly, I think, in New Jersey, where the plant is called "Water Brash."

After proceeding thus far, it was originally my intention to have offered some speculations of my own concerning the final object of Nature in constructing VEGETABLE MUSCICAPÆ, and especially those which I have ventured to call *irritable Muscicapæ*. But the present essay has already been extended to too great a length. My intended speculations may possibly form the subject of a future communication. In the mean while, these "Desultory Observations" may perhaps contribute to the amusement of some of my brother naturalists, both in Europe and in America. If they shall produce this effect, my principal object in laying them before the public, in their present crude and irregular state, will have been accomplished.

BENJAMIN SMITH BARTON, M.D.

*Philadelphia*, *Sept.* 3, 1811. *(Phil. Mag.)*

* I do not know whether this singular plant (*Aquarium sitiens* of Leschenault), belonging to the natural order of *Succulentæ*, has yet been publicly figured or described by any botanist. I have seen a drawing of the plant, which is a native of Australasia, in the possession

